# Virtual Reality Exposure Therapy for Childhood Trauma: Narrative Review of Clinician-Guided VR Recreation to Process Traumatic Events

**DOI:** 10.1192/j.eurpsy.2026.11341

**Published:** 2026-07-31

**Authors:** M. Rocha, V. Sharma, S. Prasad, S. Gunturu

**Affiliations:** 1 American University of Caribbean School of Medicine, Cupecoy, Sint Maarten (Dutch Part); 2 Dayanand Medical College and Hospital, Ludhiana, India; 3 Psychiatry, BronxCare Health System, Bronx, United States

## Abstract

**Introduction:**

Virtual Reality Exposure Therapy (VRET), a promising integration of technology and psychotherapy, enables clinicians to safely use controlled simulations of traumatic environments for therapeutic processing. Traditional trauma-focused therapies often face developmental barriers, avoidance, or emotional overload, particularly in childhood trauma. VRET provides a structured, modifiable, clinician-guided setting that engages the sensory and emotional components of trauma while overcoming key limitations of conventional approaches.

**Objectives:**

We explore the therapeutic, neurobiological, and ethical foundations of VRET for childhood trauma through a narrative review, emphasizing how immersive environments can safely evoke traumatic memory reconsolidation and emotional regulation under clinician supervision. The review aims to identify key design principles for developmental appropriateness, therapeutic pacing, and trauma-informed safety in VRET.

**Methods:**

A comprehensive literature search was conducted in **PubMed**, **Scopus**, and **PsycNet** using the keywords *“Virtual Reality,” “Virtual Exposure Therapy,” “Childhood Trauma,”* and *“Adverse Childhood Events,”* with no temporal or geographical restrictions. After removing duplicates, studies were screened by title and abstract; relevant English-language original and review articles were included. **Non-English publications, commentaries, editorials, and opinion papers** were excluded. Additional references were identified through **citation screening** of key studies and a **targeted open-source Google search** to capture conceptually relevant material.

**Results:**

Recent studies indicate that VRET reduces post-traumatic stress, anxiety, and dissociation while maintaining psychological safety. In a randomized trial (n=72) with childhood trauma, participants showed significant improvements in depressive and trauma scores (*p* < 0.05) sustained for three months. A second trial (n=44) with childhood sexual abuse replicated these findings but found no remission difference versus standard therapy. Beyond treatment, VR aids diagnosis by assessing stress reactivity, psychosis vulnerability, and maladaptive schemas, though ethical and neurodevelopmental effects on memory and resilience remain underexplored.

**Conclusions:**

Virtual Reality Exposure Therapy holds transformative potential in childhood trauma care, enabling clinicians to safely simulate and recontextualize traumatic environments within a framework of trust and therapeutic containment. By bridging neuroscience, technology, and developmental psychology, VRET may accelerate trauma processing and resilience-building in ways previously unattainable in traditional talk-based therapy. Ethical oversight, individualized calibration, and clinician training will be essential to ensure safety and emotional integrity as this technology moves toward broader clinical implementation.

**Disclosure of Interest:**

None Declared

